# Severe clinical relapse in an immunocompromised host with persistent SARS-CoV-2 infection

**DOI:** 10.1038/s41375-021-01175-8

**Published:** 2021-02-19

**Authors:** Philipp A. Reuken, Andreas Stallmach, Mathias W. Pletz, Christian Brandt, Nico Andreas, Sabine Hahnfeld, Bettina Löffler, Sabine Baumgart, Thomas Kamradt, Michael Bauer

**Affiliations:** 1 Department of Internal Medicine IV (Gastroenterology, Hepatology and Infectious Diseases), Jena University Hospital, Jena, Germany; 2 Institute for Infectious Diseases and Infection Control, Jena University Hospital, Jena, Germany; 3 Institute for Immunology, Jena University Hospital, Jena, Germany; 4 Praxis für Onkologie, Ambulantes Medizinisches Zentrum Jena GmbH, Jena, Germany; 5 Institute for Medical Microbiology, Jena University Hospital, Jena, Germany; 6 Core Facility Cytometry, Institute for Immunology, Jena University Hospital, Jena, Germany; 7 Department of Anesthesiology and Intensive Care Medicine, Jena University Hospital, Jena, Germany

**Keywords:** Lymphoma, Infectious diseases

## To the Editor:

Whether people who have recovered from COVID-19 can be re-infected by SARS-CoV-2 is a matter of debate. Antibodies against SARS-CoV-2 can be detected in up to 98.6% of patients after infection, but only in 67% of patients with CLL [1]. In this context, anti-CD20 therapy is of special interest, as the memory B-cells are crucial for the development of immunity against SARS-CoV-2 [2]. A recent study from China has revealed that the failure to mount a robust humoral response against SARS-CoV-2 is associated with re-detection of SARS-CoV-2 in 7.3% of patients [3]. In addition, patients with hematological malignancies are also more vulnerable to a severe course [4].

In this brief report, we present the case of a female patient with a rituximab-treated B-cell lymphoma with severe relapse 4 months after moderate COVID-19 due to SARS-CoV-2.

We present the case of a 56-year old woman who was diagnosed with a follicular lymphoma (grade 2–3a, stage IV, bone marrow >50%) in 2019. The patient was started on a combination therapy with rituximab (375 mg/m^2^) and bendamustine (90 mg/m^2^) for six cycles, followed by single-agent rituximab (375 mg/m^2^ IV every 8 weeks) after achieving complete remission. The last treatment occurred on March 16th. On April 2nd, she developed a 38.4 °C fever without other symptoms. After 10 days, she felt shortness of breath and dry cough, and she was admitted to the hospital. A low-dose-CT chest scan revealed ground glass opacities. RT-PCR SARS-CoV2 testing on a nasal-throat-swab was positive. Physical examination revealed a body temperature of 37.9 C, a blood pressure of 126/80 mmHg, a pulse of 118 beats/min and a respiratory rate of 26 breaths/min. Her oxygen saturation remained above 93% with low flow oxygen supply. Decreased counts of white blood cells (4.2 × 10^9^/L) and lymphocytes (0.53 × 10^9^/L) were detected. The absolute counts of T-lymphocytes (CD3^+^/CD4^+^: 82 cells/μL; CD3^+^/CD8^+^: 89 cells/μL; CD4/CD8-ratio: 0.92) were decreased, and notably, no B-cells were detectable. Six days after hospitalization, she had no further symptoms. RT-PCR tests for SARS-CoV-2 remained negative, and the patient was discharged from the hospital. At 3 weeks after discharge, a SARS-CoV-2-antibody ELISA was negative (See Supplementary material).

Unexpectedly, 4 months later, her symptoms of dry cough and intermittent fever re-appeared. Outpatient care with two nasal-throat swabs revealed negative RT-PCR results. However, her fever increased, and she developed fatigue and was re-admitted to the hospital. Her chest CT showed radiographic signs indicative of COVID-19. Three SARS-CoV-2 RT-PCR tests of nasal-throat swabs and induced sputum remained negative. Despite previous infection, her SARS-CoV2-IgG was below the lower limit of detection.

In the following days, her respiratory situation detoriated, and she was transferred to the ICU. Her respiratory support needed to be switched to invasive ventilation. The patient had a Horowitz-Index of 150 and required norepinephrine support (0.05 µg/kg/min). Because the pathogen remained unknown, a bronchoscopy was performed directly after intubation, and broncho-alveolar lavage finally confirmed the presence of SARS-CoV2-RNA. Remdesivir and convalescent plasma (initially after PCR results were obtained and again 3 days later) and finally Infliximab (5 mg/kg body weight) were administered. This therapy resulted in an improvement in her condition, and extubation was achieved after 5 days of ventilation. Notably, after application of reconvalescent plasma, the patient had detectable levels of SARS-CoV2-IgG (12.6 AU/ml).

Given the suspected relapse of the SARS-CoV-2 infection, a molecular and immunological work-up was performed. Since a SARS-CoV-2 positive swab of the first episode was stored in our biobank, we were able to sequence and compare both samples.

The vi059641(t0) and vi059641(t1) genomes, both isolated 4 months apart, differed by 12 base-pair substitutions. This number of substitutions is expected after 4 months, according to the estimated annual evolution rate of 1.24 × 10^−3^ substitutions per site for SARS-CoV-2 [5, 6]. In addition, both isolates were of lineage B.1 [7] and were found to share the same origin when plotted on a phylogenetic tree with 354 other German SARS-CoV-2 isolates (Supplementary Fig. S1). Both the placement within the phylogenetic tree and the number of substitutions convincingly indicated that both isolates belonged to the same SARS-CoV-2 strain. These results suggest, that the virus has persisted and evolved within the patient during the last four months.

**Fig. 1:**
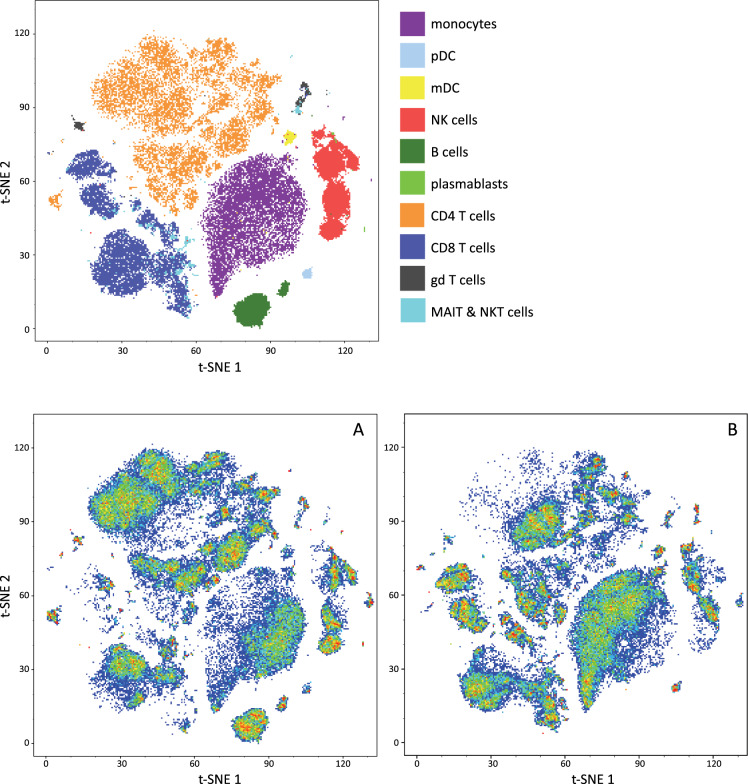
t-SNE map of blood leukocytes after exclusion of granulocytes. Cells were stained with 30 antibodies for cell surface molecules and analyzed by mass cytometry. Main leukocyte populations were assigned according to their marker expression (Supplementary Table 2) pDC: plasmacytoid dendritic cells, mDC: myeloid dendritic cells. The t-SNE maps **A** (controls) and **B** (patients) are colored according to their cell densities (red indicates the highest cell density). A complete absence of B cells in the patient is shown.

We used flow cytometry to characterize major leukocyte subpopulations in the patient and in five sex- and age-matched controls (antibody panel is presented in Supplemantary Table S1, markers used for phenotyping characterization are presented in Supplementary Table S2). As expected, the patient’s lymphocyte count was severely diminished. For detailed analyzes, we used mass cytometry and performed t-distributed stochastic neighbor embedding (viSNE) [8] to compare cell population densities in the patient and the controls (Fig. 1).

**Fig. 2:**
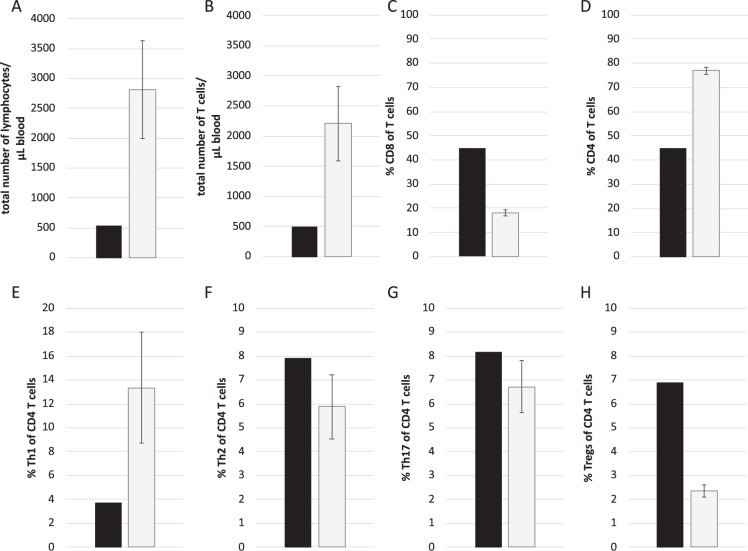
Analysis of leukocyte subpopulations of the patient. **A**–**D** Absolute numbers of CD3 + T cells and percentages of CD3 + CD8 + cytotoxic T cells and CD3 + CD4 + T helper (Th) cells in the blood of the patient (black bars) and six controls (gray bars). **E**–**H** Frequencies of Th1 (CXCR3^+^CCR6^−^CCR4^−^CXCR5^−^), Th2 (CXCR3^−^CCR6^−^CCR4^+^ CXCR5^−^), Th17 (CXCR3^−^CCR6^+^CCR4^+^CXCR5^−^), and Treg (CD25^+^CD127^−^CCR4^+^HLA-DR^−^) cells among CD3^+^CD4^+^ T cells in the patient (black bars) and controls (gray bars).

Analysis of 35 different leukocyte subpopulations revealed that the patient’s lymphopenia was marked by the complete absence of B-cells and a severe decrease in T-cells. The frequencies of γ/δ T-cells, MAIT, and NKT-cells were all lower in the patient than the controls (data not shown). In contrast to recent reports on COVID-19 [9], the percentage of CD8 + cytotoxic T-cells among all T-cells was markedly elevated in the patient, whereas the percentage of CD4 + T helper (Th) cells was diminished (Fig. 2A–D).

The functional differentiation of Th-cells revealed a markedly lower frequency of Th1-cells and a higher frequency of regulatory T-cells in the patient than in the controls. The frequencies of Th2- and Th17-cells were similar in the patient and controls (Fig. 2E–H).

The patient was discharged without residual symptoms 3 weeks after the second admission. At 6 weeks after discharge, the patient remains in good general condition, the pulmonary infiltration was completely resolved. SARS-CoV2 PCR from broncho-alveolar lavage was negative, and the SARS-CoV-2 antibodies had decreased to 4.1 AU/ml, thus suggesting that in our patient only transfused antibodies without endogenous production were detected.

In conclusion, this is the first report that has demonstrated re-activation by sequencing and applying the annual evolution rate. This case shows, that patients with impaired lymphycte populations are of higher risk for persistence and clinical reactiviation of SARS-CoV-2, especially if they do not develop anti-SARS-CoV-2 antibodies. A possible treatment strategy for these patients may be infusion of convalescent plasma. Hematologic therapies should be selected with caution, particularly those containing anti-CD20 antibodies [10].

## Supplementary information

Supplemental Material

Figure S1
