# Supplementary material for: Severe clinical relapse in an immunocompromised host with persistent SARS-CoV-2 infection
Source: Leukemia. 2021 Feb 19;35(3):920–3. doi: 10.1038/s41375-021-01175-8 (PMC7893131; doi:10.1038/s41375-021-01175-8)
Supplement: Supplementary file 1 — Supplemental Material [file 41375_2021_1175_MOESM1_ESM.docx]

**Severe Clinical Relapse in an Immunocompromised Host With Persistent SARS-CoV-2 Infection – Supplementary Material**

**Short title: COVID-19 relapse**

Philipp A. Reuken^1^ philipp.reuken@med.uni-jena.de

Andreas Stallmach^1^ andreas.stallmach@med.uni-jena.de

Mathias W. Pletz^2^ mathias.pletz@med.uni-jena.de

Christian Brandt^2^ Christian.brandt@med.uni-jena.de

Nico Andreas^3^ nico.andreas@med.uni-jena.de

Sabine Hahnfeld^5^ Hahnfeld@amz-jena.de

Bettina Löffler^6^ [Bettina.loeffler@med.uni-jena.de](mailto:Bettina.loeffler@med.uni-jena.de)

Sabine Baumgart^3,4^ sabine.baumgart@med.uni-jena.de

Thomas Kamradt^3,4^  thomas.kamradt@med.uni-jena.de

Michael Bauer^7^ Michael.bauer@med.uni-jena.de

All authors declare no conflict of interest.

**Affiliations:**1: Department of Internal Medicine IV (Gastroenterology, Hepatology, and Infectious Diseases), Jena University Hospital, Jena, Germany

2: Institute for Infectious Diseases and Infection Control, Jena University Hospital, Jena, Germany

3: Institute for Immunology, Jena University Hospital, Jena, Germany

4: Core Facility Cytometry, Institute for Immunology, Jena University Hospital, Jena, Germany

5: Praxis für Onkologie, Ambulantes Medizinisches Zentrum Jena GmbH, Jena, Germany

6: Institute for Medical Microbiology, Jena University Hospital, Jena, Germany

7: Department of Anesthesiology and Intensive Care Medicine, Jena University Hospital, Jena, Germany

**Corresponding author:**Philipp Reuken, Department of Internal Medicine IV, Jena University Hospital, Friedrich Schiller University of Jena, Am Klinikum 1, 07747 Jena, Germany. Fax: +49-3641-9324222, Email: [philipp.reuken@med.uni-jena.de](mailto:philipp.reuken@med.uni-jena.de)

**Word count: 239**

**References: 2**

**Figures: 3**

**Materials and Methods**

Sequencing and genome reconstruction

Library preparation was performed according to the 'nCoV-2019 sequencing protocol' (dx.doi.org/10.17504/protocols.io.bdp7i5rn) from the ARTIC network (https://artic.network/ncov-2019). Briefly, viral RNA was isolated for both SARS-CoV-2 virus strains via the QIAmp viral RNA Kit (Qiagen, Hilden, Germany) according to the manufacturers' guide. The cDNA preparation was performed using SuperScript IV (Thermofisher), followed by a multiplex PCR to generate overlapping 400 nt amplicons using version 3 of the ARTIC primer set (https://github.com/artic-network/artic-ncov2019/tree/master/primer_schemes/nCoV 2019/V3). After PCR cleanup, library preparation was performed using the Ligation Sequencing Kit (LSK-109, Oxford Nanopore Technologies) and the Native Barcoding Expansion (EXP-NBD104, native Barcoding Kit (Oxford Nanopore Technologies)). Sequencing was performed on a MinION device using an R.9.4.1 flow cell (Oxford Nanopore Technologies). Basecalling and genome reconstruction was performed using poreCov v0.2 with the default settings (https://github.com/replikation/poreCov)^1^.

Time tree construction and strain comparison

Strains were directly compared via blastn (default settings) to identify nucleotide differences. The time tree was constructed via additionally adding 354 German SARS-CoV-2 strains from GISAID (downloaded August 2020) and computed and plotted via augur v6.4.3 (https://github.com/nextstrain/augur) and toytree v1.1.2^2^ using the poreCov v0.2 workflow. SARS-CoV-2 Lineage was determined using pangolin v2.0.4 (<https://github.com/cov-lineages/pangolin>).

Multiparameter single cell analysis of peripheral blood leukocytes using mass cytometry

The female patient of the study was 56 years old. Five gender and age matched healthy donors (average 55 *± 2* years) were included as control group from Neustadt Study. After venesection, heparinized blood was used to determine the absolute number of leukocytes by using Trucount^TM^ Beads (BD Biosciences, San Jose, CA, USA) and flow cytometry (BD FACSCanto Plus Cell Analyzer). For high dimensional immune profiling of peripheral blood leukocytes a commercially available 30-marker antibody panel (Supplementary Table S1) was used (Maxpar® Direct^TM^ Immune Profiling System (Fluidigm Inc., Canada) to stain whole blood samples. Briefly, 270 µL blood were treated with heparin (heparin sodium salt from procine intestinal mucosa, #H3149-25KU, Sigma Aldrich) blocking reagent 30 µL of 1,000 U/mL heparin in PBS, incubation at room temperature (RT), 20 min) and added after then into a tube containing the lyophilized antibody cocktail (incubation at RT, 30 min). After then, red blood cells were lysed using 250 µL CAL-Lyse Lysing Solution (Life Technologies, Rockville, MD, USA) (incubation at RT, 10 min, dark). 3 mL Millipore Q water were added and further incubated (RT, 10 min, dark). After centrifugation (300 g) and washing the cells three times with MaxPar Cell staining buffer, cell pellet was resuspended and fixed with 1 mL 1.6 paraformaldehyd (EMS, Hatfield, PA, USA) (RT, 10 min). After centrifugation (800 g) cells were incubated with 1 mL Maxpar Fix and Perm Buffer with 1:1,000 iridium (Cell-ID DNA intercalator, 125 µM (4°C, 2 h). Cells were centrifuged (800 g) and 900 µL supernatant were discarded. The rest was stored at -80°C until mass cytometric measurement (CyTOF, Cytometry time of flight).

Before measurement, cells were thawed, washed twice with 2 mL MaxPar Cell staining buffer, and twice with Cell acquisition solution. Cells were counted using a hemacytometer and adjusted to 7.5 x10^5^ cells/mL. 10% v/v Four Element calibration beads (Fluidigm) as internal standard beads were added before acquisition of an average of 500,000 events using the Helios mass cytometer equipped with CyTOF software v7.0.8496.0 (Fluidigm). FCS data files were normalized based on signals of the internal standard beads. Data analysis were performed using MaxPar Pathsetter software v2.x to obtain frequencies of 35 leukocytic cell subsets for immune profiling (Supplementay Table 2). FlowJo v10 (BD) was used for manual gating and t-distributed stochastic neighbor embedding (t-SNE) map generation. Pre-cleaned files from Pathsetter sofware were used to manually remove granulocytes (neutrophils, basophils and eosinophils). Subsequently, the t-SNE map was generated to visualize all lymphocyte populations by using the following settings: iterations 1000, perplexity 30, vantage point tree k-nearest neighbors algorithm and Barnes-Hut gradient algorithm. For better comparison of t-SNE maps from patient and the healthy control group, the total number of cells were normalized to an equal number of 95,600.

**Literature**

1. Hufsky, F. *et al.* Computational Strategies to Combat COVID-19: Useful Tools to Accelerate SARS-CoV-2 and Coronavirus Research. (2020) doi:10.20944/preprints202005.0376.v1.

2. Eaton, D. A. R. Toytree: A minimalist tree visualization and manipulation library for Python. *Methods Ecol. Evol.* **11**, 187–191 (2020).

**Tables and Figures:**

| Specificity | Clone | Isotope |
| --- | --- | --- |
| CD45 | HI30 | ^89^Y |
| CD196/CCR6 | G034E3 | ^141^Pr |
| CD123 | 6H6 | ^143^Nd |
| CD19 | HIB19 | ^144^Nd |
| CD4 | RPA-T4 | ^145^Nd |
| CD8a | RPA-T8 | ^146^Nd |
| CD11c | Bu15 | ^147^Sm |
| CD16 | 3G8 | ^148^Nd |
| CD45RO | UCHL1 | ^149^Sm |
| CD45RA | HI100 | ^150^Nd |
| CD161 | GP-3G10 | ^151^Eu |
| CD194/CCR4 | L291H4 | ^152^Sm |
| CD25 | BC96 | ^153^Eu |
| CD27 | O323 | ^154^Sm |
| CD57 | HCD57 | ^155^Gd |
| CD183/CXCR3 | G25H7 | ^156^Gd |
| CD185/CXCR5 | J252D | ^158^Gd |
| CD28 | CD28.2 | ^160^Gd |
| CD38 | HB-7 | ^161^Dy |
| CD56/NCAM | NCAM16.2 | ^163^Dy |
| TCRgd | B1 | ^164^Dy |
| CD294 | BM16 | ^166^Er |
| CD197/CCR7 | G043H7 | ^167^Er |
| CD14 | 63D3 | ^168^Er |
| CD3 | UCHT1 | ^170^Er |
| CD20 | 2H7 | ^171^Yb |
| CD66b | G10F5 | ^172^Yb |
| HLA-DR | LN3 | ^173^Yb |
| IgD | IA6-2 | ^174^Yb |
| CD127 | A019D5 | ^176^Yb |
| Live/dead –  DNA intercalator |  | ^103^Rh  ^191^Ir/^193^Ir |

**Supplementary Table 1 Maxpar Direct Immune Profiling Assay Panel**

**Supplementary Table 2 Phenotypic characterization of lymphocytic cell subsets**

Pre-gating was performed using Pathsetter software to obtain single viable CD40-Ir+ CD45+ leukocytes according to the technical note. Granulocytes were manually removed by excluding following gates CD66b+CD16+ (neutrophils) and CD66b+CD16dim (eosinophils) followed by HLA-DR-CD123+CD38+ (basophils).The following marker expressions were used to assign lymphocytic cell subsets.

| B cells | CD3-CD14-CD19+CD20+HLADR+ |
| --- | --- |
| Plasmblasts (PB) | CD3-CD14+CD19+CD20-CD38++CD27++ |
| monocytes | CD19-CD20-CD3-CD56-CD11c+/lowHLA-DR+ |
| Dendritic cells (DC)  pDC  mDC | CD3-CD19-CD14-CD20-HLA-DR+CD11c-CD123+  CD3-CD19-CD14-CD20-HLA-DR+CD11c+CD123-CD16-CD38+CD294- |
| Natural Killer cells (NK) | CD14-CD3-CD19-CD20-CD123-CD45RA+CD56+ |
| CD 8 T cells (cytotoxic T cells) | CD3+CD19-CD20-CD4-CD14-CD8+ |
| CD 4 T cells (T helper cells) | CD3+CD19-CD20-CD8-CD14-CD4+ |
| activated CD 8 T cells | CD3+CD19-CD20-CD4-CD14-CD8+CD38+HLA-DR+ |
| activated CD 4 T cells | CD3+CD19-CD20-CD8-CD14-CD4+CD38+HLA-DR+ |
| gd T cells | CD3+CD14-CD8dim CD4-TRCRgd+ |
| Mucosal-associated invariant T (MAIT) and NKT cells | CD3+CD4-CD14-CD161+CD28+ |
