# Supplementary figures and images for: Severe clinical relapse in an immunocompromised host with persistent SARS-CoV-2 infection
Source: Leukemia. 2021 Feb 19;35(3):920–3. doi: 10.1038/s41375-021-01175-8 (PMC7893131; doi:10.1038/s41375-021-01175-8)

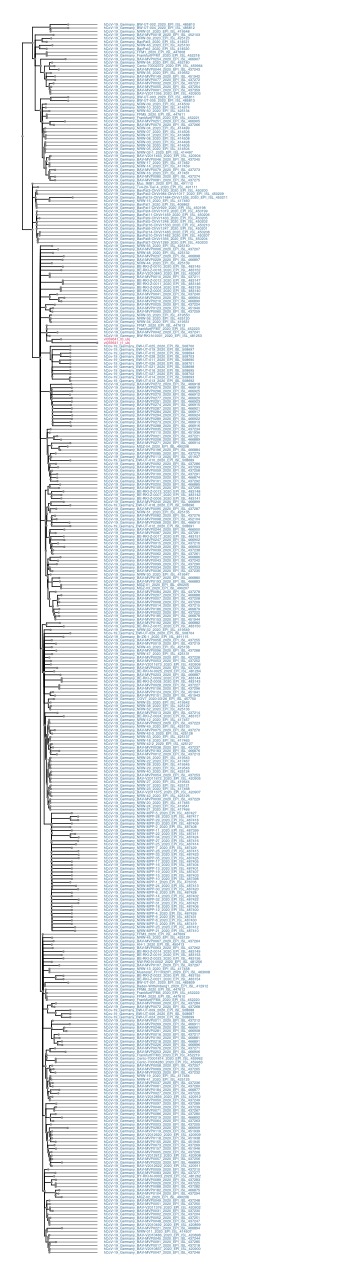

Supplement: Supplementary file 2 — Figure S1 [file 41375_2021_1175_MOESM2_ESM.jpg]
